# Barn Owl Productivity Response to Variability of Vole Populations

**DOI:** 10.1371/journal.pone.0145851

**Published:** 2015-12-28

**Authors:** Petr Pavluvčík, Karel Poprach, Ivo Machar, Jan Losík, Ana Gouveia, Emil Tkadlec

**Affiliations:** 1 Department of Ecology and Environmental Sciences, Faculty of Science, Palacky University Olomouc, Olomouc, Czech Republic; 2 TYTO–občanské sdružení, Nenakonice, Věrovany, Czech Republic; 3 Department of Development Studies, Faculty of Science, Palacky University Olomouc, Olomouc, Czech Republic; 4 Institute of Vertebrate Biology Academy of Sciences of the Czech Republic, Brno, Czech Republic; University of Lausanne, SWITZERLAND

## Abstract

We studied the response of the barn owl annual productivity to the common vole population numbers and variability to test the effects of environmental stochasticity on their life histories. Current theory predicts that temporal environmental variability can affect long-term nonlinear responses (e.g., production of young) both positively and negatively, depending on the shape of the relationship between the response and environmental variables. At the level of the Czech Republic, we examined the shape of the relationship between the annual sum of fledglings (annual productivity) and vole numbers in both non-detrended and detrended data. At the districts’ level, we explored whether the degree of synchrony (measured by the correlation coefficient) and the strength of the productivity response increase (measured by the regression coefficient) in areas with higher vole population variability measured by the *s*-index. We found that the owls’ annual productivity increased linearly with vole numbers in the Czech Republic. Furthermore, based on district data, we also found that synchrony between dynamics in owls’ reproductive output and vole numbers increased with vole population variability. However, the strength of the response was not affected by the vole population variability. Additionally, we have shown that detrending remarkably increases the Taylor’s exponent *b* relating variance to mean in vole time series, thereby reversing the relationship between the coefficient of variation and the mean. This shift was not responsible for the increased synchrony with vole population variability. Instead, we suggest that higher synchrony could result from high food specialization of owls on the common vole in areas with highly fluctuating vole populations.

## Introduction

In recent years, ecologists have increasingly recognised the importance of environmental variability on population growth rate, behavioural strategies and individual fitness [1, 2]. The non-linear responses of organisms to environmental variability are prevalent in biology. In a fluctuating environment, their long-term response measured as the average in a long run is inevitably either higher or lower than that measured in a less variable or constant environment. This is a direct result of Jensen’s inequality, a mathematical property stating that for a non-linear function *f*(*x*) the average function $\bar{f(x)}$ does not equal to $f(\bar{x})$ (see [3] or [4] for detailed graphical explanation). Based on this rationale, life history theory predicts that temporal environmental variability in food resource availability can affect the long-term organism’s response (e.g., production of young) positively if the function is accelerating (convex up, the second derivative is positive), i.e., organisms respond to environmental stochasticity by slightly decreasing productivity in periods of low food abundance but greatly increasing productivity in good years. Strategies that increase this convexity are favoured by natural selection as they lead to higher fitness [3, 4]. On the other hand, when productivity response is a concave function of a stochastically fluctuating resource, strategies that decrease the concavity are favoured by avoiding the detrimental effects of bad years. Organisms, thus respond evolutionarily to low food abundance by substantially decreasing their breeding capacity which in years of high food abundance becomes saturated and allows only for slightly increased productivity. This saturating response can be expected in some avian predators nesting once a year with a limited capacity to increase the clutch size in years of high food availability [4, 5]. There is no effect of environmental variability on a long-term productivity average if the function is linear, or the effect can further complicate if the underlying function is sigmoid [6].

Birds and mammals feeding on small herbivore voles whose population numbers fluctuate largely in time represent a useful model system to test this theory. Vole dynamics typically consist of long intervals of low abundance and short intervals of superabundance [7]. This is why the voles are commonly considered a good example of environmental stochasticity referred to as pulsed resources [8–10]. Annual productivity of vole-hunting animals has been documented to decrease in years of low vole abundances in both avian and mammalian predators. Such reports on their breeding responses include the significant decrease in clutch sizes in the hen harrier [11], the tawny owl [12], and the Ural owl [13, 14], the reduced number of fledglings in the barn owl [15], or reduced egg size and hatching success in the Eurasian kestrel [16]. In mammals, arctic foxes decrease ovulation rate [17] resulting in smaller litter sizes and productivity [18, 19]. Weasels have also been observed to lower embryo and offspring survival rates [20].

Unlike evidence on how these predators respond to changes in resource availability, we are much less informed on how they respond to changing variability in resource availability. Hušek et al. [5] examined the white stork–common vole system and concluded that breeding responses of storks, measured as cross-correlation between annual stork productivity and vole abundances, were stronger in areas with higher variability of vole numbers. Interestingly enough, the relationship between the numbers of fledglings and vole abundances was observed to be concave, thereby predicting the opposite. One way of reconciling the theory with this odd observation is that the strength of the breeding response was erroneously estimated using cross-correlations. Cross-correlations can give us an estimate of closeness of points to a linear relationship or temporal synchrony between productivity and vole numbers but not the rate of change in productivity as a function of changes in vole numbers. Regression coefficients do that, hence these measure the strength of the stork breeding response to vole variability more appropriately and accurately. Another potential explanation for the positive relationship between the breeding response and population variability has been suggested by Barraquand and Hušek [4] who found an exceptionally high exponent *b* from Taylor’s law describing the dependence of variance on the population mean in the vole population index. Their best-fit estimate of *b* for the Czech and Polish populations was found to be somewhere between 4 and 6, thereby deviating greatly from the interval of 1 to 2 observed for most organisms [21]. As a result, voles’ population variability increases with the population mean. The positive effect of variation in vole numbers on the strength of response then arises from its covariation with mean vole density. To assess the generality of previous studies, analyses of further empirical cases are therefore warranted.

Here we analyse the barn owl–common vole system to study the responses of the barn owl annual productivity to common vole population numbers and variability in central Europe. By applying two different methodological approaches, one with non-detrended original data and the other with detrended data prior to analysis, we then described the functional relationship between the annual sum of fledglings and vole numbers at the level of the whole Czech Republic. Next we focus on the variation in annual productivity relative to the vole population variability using data from ten Czech districts. Addressing the main question: when responding to the same change in vole numbers, do the owls increase or decrease the number of fledglings equally in all areas irrespective of vole population variability? To describe the strength of the responses in annual productivity, we use the slope estimate from a linear regression of annual productivity on the vole numbers in the 10 districts. Finally, we demonstrate how detrending the time series can shift Taylor’s exponent and potentially divert ecological inference.

## Methods

### Barn owl data

The barn owl (*Tyto alba*) is a long-lived medium-sized owl with high breeding site fidelity [22, 23]. They typically prefer farm buildings and church towers as nesting sites and their home range covers an area of 5 to 10 km^2^ [24]. This species nests from April to July and occasionally nests twice or three times a year [25]. In open farmlands small rodents are crucial preys constituting up to 90% of their diet [24]. The common vole (*Microtus arvalis*) is the most common food item out of all small rodents [26]. Other preys like birds, amphibians and insects are of reduced importance. Obvious prey switching from mammals to other prey classes does not take place even when small mammals decrease to 7% of the available food, suggesting that barn owls are highly specialized predators of small rodents [27].

**Fig 1:**
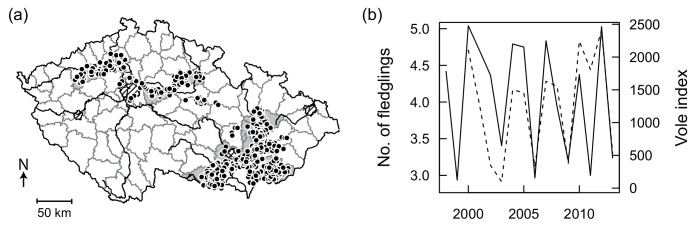
(a) Map of the districts in the Czech Republic showing the distribution of barn owl nesting sites and (b) the dynamics of barn owl productivity (solid lines) and the common vole numbers (dashed line) in autumn during the period 1998–2013. The shaded areas in (a) indicate the 10 districts used in the analysis of vole population variability effects on the strength of the responses in the barn owl productivity parameters. Barn owl productivity was measured as the annual number of successfully produced fledglings (solid line). Vole numbers were measured by a vole index based on the number of active burrow entrances per hectare.

The fledglings remain dependent on their parents for one to two months. In Western Europe, mainly juvenile barn owls were able to migrate over large areas and follow places of high prey abundance [28]. The loss of suitable nesting sites has been suggested as one of the main causes of population decline in Western Europe [24, 29]. To support the barn owl population in the Czech Republic, 1470 artificial nest-boxes specifically designed to protect young against predators were installed in farm buildings between 1996 and 2001. Within few years of the national monitoring and ringing programme, these nest-boxes have become widely used by owls. Between 1998 and 2013, we monitored the barn owls’ nesting population by checking all nest-boxes. Additionally, we also searched for natural nests in suitable habitats, such as sacral buildings, castles and farm buildings. All known nesting sites were checked at least once a year in late May to July. The occupied nests were then repeatedly visited for ringing of young and in autumn to check the second nesting attempt. On rare occasions, nesting was delayed until August. These cases were identified during the following year control by signs such as pellets or abandoned eggs left by. In total, 662 potential nesting sites were examined regularly every year during 1998–2013. Each occupied nesting site was visited repeatedly over the whole nesting period between May and November and the number of eggs, hatchlings and fledglings were recorded, these data include direct observations of eggs, hatchlings or fledglings but also back calculations from nestlings and unhatched eggs (about 40%). In total, we collected 1667 observations on the number of eggs, 1580 observations on the number of successful hatchlings, and 1579 observations on the number of successful fledglings from 681 different nesting sites situated in 17 Czech districts (Fig 1). In our sample, the second and third nesting attempts were recorded in 31.7% and 0.3% nesting sites, respectively, similar to reported data from Germany [30]. We assume that the second and third clutches were produced by the same female due to evidence of very high site fidelity in adult barn owls [22, 23].

### Ethics statement

The ringing data on barn owls were collected in accordance with ethical standards following the Act No. 418 246/1992 Coll. on the protection of animals against cruelty by KP and IM who are official bird ringers in the Czech Republic. They have authorisation by the Ministry of the Environment of the Czech Republic to research and protect the Western Barn Owl (No. 6826/01–OOP/2110/01–V847) and after revision of the jurisdiction the permission was issued by the Nature Conservation Agency of the Czech Republic No. 426/PA/2007. They also hold the permission issued by Prague Ringing Centre No. 804to ring birds such as the Western Barn Owl. The areas in the open agricultural land are freely accessible in the Czech Republic and no permission is required. We co-operated with hundreds of agricultural building’s owners (mostly farmers). And we have written agreements from all of the owners allowing us to enter the agricultural buildings set up nest-boxes and carry out the research.

### Common vole data

The common vole (*Microtus arvalis*) is the most abundant microtine rodent in central European farmlands primarily inhabiting open grassy habitats and agricultural fields with forage crops, such as alfalfa and clover. Their abundances fluctuate strongly with peaks occurring at intervals of three to four years [7]. The peculiar feature of this species is that autumn local population densities can attain more than two thousand individuals per hectare in peak years [31], whereas during population lows the numbers may decrease to virtually zero. The common vole population densities in this study were assessed at the district level, administrative units of on average 1000 km^2^ in size, using a population index based on counts of active burrow entrances per hectare. These are indicated by the presence of signs, such as smooth margins of the entrances, fresh plants placed inside the burrow openings, fresh heaps of soil and/or fresh droppings. Since 2000, the State Phytosanitary Administration has monitored its numbers in various crops in the Czech Republic twice a year during spring (March–April) and autumn (October–November). Here we use data collected exclusively in fodder, such as alfalfa, clover, or cultivated meadows known as the common vole preferred habitats. In each district, 10 sites were surveyed for the number of active burrow entrances by walking along four 100-m strips, each 2.5 m wide. The counts collected on a total area of 1000 m^2^ were then multiplied by 10 to obtain the numbers per hectare.

### Statistical analysis

We analysed the relationship between the barn owl reproduction and vole abundances at the level of the Czech Republic. The data on barn owl productive output and vole numbers are time series. Temporal trends in non-stationary series may present a problem not only when measuring population variability [32] but also when measuring the relationships between the two time series by causing spurious correlations due to correlated trends [33]. Since ecologists are most often interested in measuring correlations for fluctuations around the trend rather than those for trends, these are routinely removed prior to analysis [34]. This approach is justified if the time series are long and trends are proven to be real at a reasonable level of certainty. However, if the time series are rather short, as with our data (14 years), and there is no external evidence of environmental change, the data are best analysed both with and without detrending in order to document the effects of detrending on the obtained results [34:176]. We, therefore, adopted two analytical approaches, one is based on the original data for barn owls and voles and another on the data with linear trends removed. Detrending of vole time series was done on a log scale (S1 Fig). The annual barn owl productivity was measured as the mean number of the successful fledglings produced per site.

We started by performing a correlation analysis to compare abilities of spring and autumn vole indices to predict the annual number of fledglings. The seasonal abundances of voles for the Czech Republic were derived as the means calculated from the district vole abundances weighted by the number of barn owl data from that district, i.e., we used the district abundances of voles in frequencies that corresponded to the numbers of barn owl measurements from that district. The annual number of clutches and annual fledgling productivity were then regressed on the annual vole abundances by fitting a weighted linear model. Reciprocals of variance for averaged annual number of clutches and annual numbers of fledglings were used as weights. Next, we analysed the relationship between the numbers of fledglings and vole numbers at the district level. We have chosen 10 districts in which the data on both owls and voles were available for more than eight years. To characterize the closeness or temporal synchrony between the dynamics of fledgling and vole numbers, we calculated cross-correlation coefficients. To quantify the strength of owls’ responses to variation in vole numbers, we used regression slopes. Standard errors for the cross-correlation coefficients were obtained by bootstrapping using 10000 randomly drawn samples. Lastly, the cross-correlation coefficients and regression slopes were modelled as a function of vole variability measured as the *s*-index, i.e., the standard deviation of log_10_-transformed vole densities. We fitted a weighted linear regression to consider the different uncertainties in parameter estimates. Reciprocals of variances were used as weights for the cross-correlation and slope coefficients. For the detrended data approach, we calculated detrended *s*-index, i.e., *s*-index based on detrended vole time series of abundances. The best-fitting model was selected according to the lowest AICc [35] from two models: intercept-only model and model containing the effect. The difference in AICc (ΔAICc) between these two models, when greater than two was considered as a strong evidence for the best model. To describe autumn vole population dynamics, specifically the cycle period, we fitted autoregressive log-linear models of order 2 using the function arima in R [36]. The model is defined by the equation (e.g., [37]):

$$X_{t}=a_{0}+\left(1+a_{1}\right)X_{t-1}+a_{2}X_{t-2}+\varepsilon_{t}$$ (1)

in which *X*_*t*_ is the logarithm of autumn vole index in a given year *t*, *ε*_*t*_ is a Gaussian noise term and *a*_1_ and *a*_2_ are the estimates of annual direct and delayed density dependence determining the length of the cycle period [38]. In particular, the more negative the parameter 1 + *a*_1_, the shorter the cycle period. Through relationships to density dependence parameters, vole population variability can be related to the variation in a cycle period, i.e., the time interval between the two population peaks. We then examined the relationship between the degree of synchrony and strength of productivity responses on density dependence parameters by fitting weighted linear regression models with direct or delayed density dependence parameters as predictors.

Lastly, to demonstrate how the detrending procedure can affect the parameter value of a Taylor’s exponent *b* and the relationship between the coefficient of variation (CV), which is another proposed measure of population variability [39], and mean population density, we computed the exponent *b* from Taylor’s power law for the variance–mean relationship Var = *a*Mean^*b*^. By taking logs, a linear functional form can be obtained

log(Var)=log(a)+blog(Mean) (2)

in which, the parameter *b* is estimated by the ordinary least square method. In a vast majority of organisms, the exponent *b* falls between 1 and 2 [32]. The Taylor’s law also determines the relationship between the CV and the mean population density:

$$\text{CV}=a^{0.5}{\text{Mean}}^{0.5b-1},$$ (3)

where, CV = (1+1/4*n*)*s*/Mean, *s* is the standard deviation of vole population numbers *N*, and *n* is the sample size. If *b* < 2, then the CV is negatively related to the population mean.

## Results

**Fig 2:**
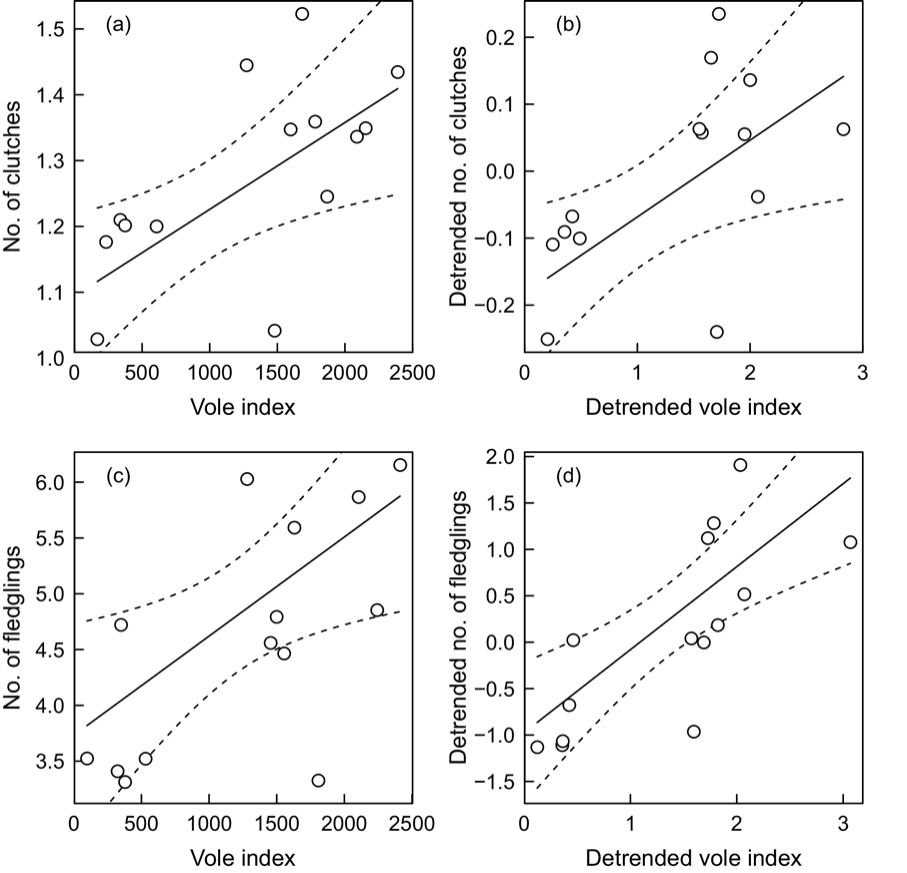
The relationship between barn owl productivity responses and autumn vole index using non-detrended (a, c) and detrended (b, d) time series, based on the data from the whole Czech Republic. The barn owl responded to the increased vole population densities by increasing the mean number of clutches per site (a, b) and the mean number of fledglings per site (c, d). The regression was weighted by reciprocals of variance for annual means of owl productivity. The dashed lines indicate 95% confidence intervals for the regression line.

The overall mean number of clutches produced each year per nesting site over the study period 1998–2013 was 1.31 (SE 0.010, n = 2071, max = 3). The overall mean number of fledglings produced per nesting site was 4.82 (SE 0.084, n = 1369, min = 0, max = 17). Based on original data, the number of fledglings per year *t* covaried less with vole spring abundances in year *t* (correlation coefficient 0.438, 95% CI −0.121 to 0.786) than autumn abundances in year *t* (correlation coefficient 0.656, 95% CI 0.192 to 0.880; Fig 1). The same was true for detrended data but the correlations were stronger (spring: 0.629, 95% CI 0.149 to 0.870; autumn: 0.754, 95% CI 0.372 to 0.917). At the country level, there was no time lag in synchrony of owl annual productivity with vole abundances. Since the annual productivity of owls was better predicted by autumn vole abundances, only autumn indices of vole numbers were used in further analyses. By applying linear regression analysis, we found evidence for the mean number of clutches and fledglings to be positively related to the number of voles in autumn for non-detrended (clutches: ΔAICc = 2.45; fledglings: ΔAICc = 10.68). For detrended data, evidence for clutches was weak (ΔAICc = 1.68) but strong for fledglings (ΔAICc = 16.12; Fig 2). There was no indication of nonlinearity in the responses of owls.

**Fig 3:**
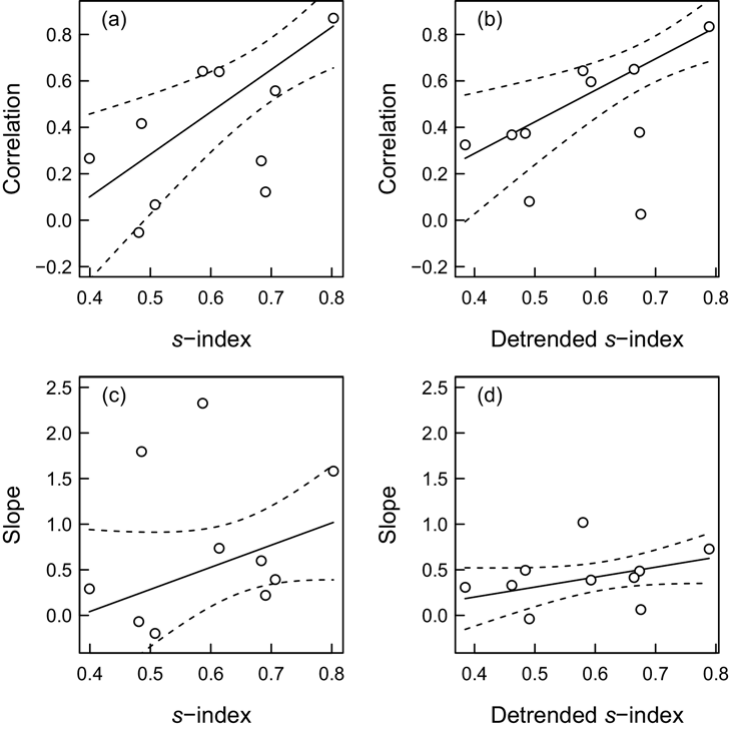
The relationship between barn owl productivity responses and vole population variability in ten districts of the Czech Republic. The upper panels show the degree of synchrony between the barn owl productivity and vole population variability using the non-detrending (a) and detrending approach (b). The lower panels show the strength of the productivity response to vole population variability without detrending (c) and with detrending (d). The dashed lines indicate 95% confidence intervals for the regression line.

At the districts’ level, we fitted weighted linear regression models to examine the relationship between temporal synchrony for barn owl productivity and vole index and the strength of the response of fledgling number to vole population variability. We obtained evidence of increased synchrony with vole population variability with both non-detrended (ΔAICc = 5.99) and detrended data (ΔAICc = 4.16; Fig 3A and 3B). However, there was no evidence for the strength of the response in the numbers of fledglings to increase with population variability, with the intercept-only models giving a slightly better fit to the data (non-detrended: ΔAICc = 1.06; detrended: ΔAICc = 0.31; Fig 3C and 3D). We also did the same analysis for the effects of vole population means in 10 districts. The only effect we have found was for detrended vole index which increased synchrony between barn owl productivity and vole index (S2 Fig).

**Fig 4:**
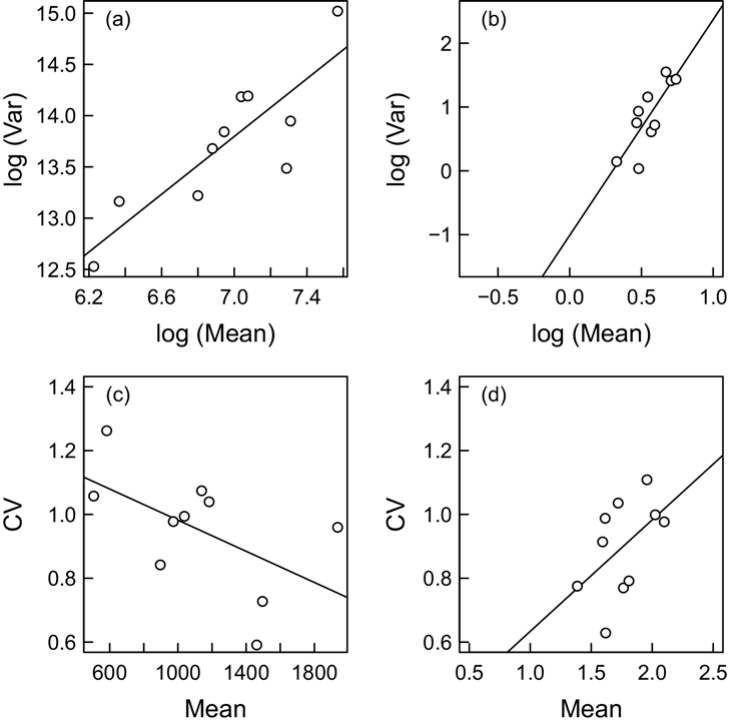
The Taylor’s power law relationships for the vole time series data. The upper panels show the relationship between variance and mean for non-detrended (a) and detrended (b) data. The lower panels show the relationship between the coefficients of variation (CV) and mean for non-detrended (c) and detrended (d) vole data.

We found no evidence for the models containing the effects of direct or delayed density dependence on synchrony or strength of the response (S3 and S4 Figs). Finally, we regressed log (Var) on log (Mean) for non-detrended and detrended vole data to check for the value of Taylor’s exponent (Fig 4). The exponents were 1.41 (95% CI 0.70 – 2.12) and 3.38 (95% CI 1.41 – 5.35) for non-detrended and detrended data, respectively. As a result of detrending, the relationship of CV to mean was reversed from a negative to a highly positive one, suggesting that the loss of variation due to detrending introduced a positive dependence of the variance on the mean.

## Discussion

While the breeding responses of most vole predators to changes in prey abundance are well documented [11–16], their responses to temporal variability in prey abundances are rare [5, 6] and poorly understood [4]. Here we examined the barn owls’ annual productivity (defined as the annual sum of fledglings produced) in relation to the common vole fluctuating populations in central Europe, specifically focusing on the effect of population variability on the strength of the productivity response. Using two analytical approaches, one with non-detrended and another with detrended data, we found that the annual number of fledglings increased linearly with autumn vole numbers. We obtained strong evidence that the degree of synchrony between the owls’ reproductive output and vole numbers increased with population variability. In contrast, the strength of productivity response did not. Moreover, we showed that detrending, substantially affected the value of the Taylor’s exponent and thus reversed the relationship between population variability and population mean. Overall, these results suggest that unlike the mean, vole population variability does not influence the productivity response in barn owls. However, they do provide supportive evidence for the findings obtained by Hušek et al. [5] with white stork–vole system that the productivity responses of consumers to the fluctuating common vole abundances are more precise in areas with more variable vole populations.

As expected, we demonstrated that the barn owls’ productivity increased with the common vole numbers. The fact that autumn vole numbers are capable of better predicting the barn owls’ productivity than spring numbers can perhaps be best explained by the owls’ ability to adjust their reproductive investments steadily over the breeding season according to changing food availability. Alongside a change in brood survival, the responses in the number of clutches and clutch sizes can lead to a close fit between overall annual productivity and vole numbers in autumn. Hence, there is no need to invoke any anticipatory responses as proposed in seed predators such as rodents ([40] but see [41]).

The observed close relationship between the owls’ reproductive output and vole numbers once again confirms the importance of the common vole as the primary food resource for barn owls in central Europe and the fact that the response of owls is quick, with no time-lag [24, 26, 28]. Although the close linkage between vole-eating birds and voles in Europe is well known, the quantitative descriptions of predators’ breeding responses to variation in prey abundance are rarely reported [30, 42, 43]. Some of them are concave, such as those for Montagu's Harrier [42], white storks [5] or long-tailed skuas [6] but some are obviously linear, such as that reported for the barn owl in Scotland [22]. In particular, the latter is in agreement with our data. This linear relationship means that owls respond to increases and decreases in vole numbers equally, i.e., the rate of change in the number of fledglings produced per unit increment in prey numbers is constant over the whole range of vole densities. Consequently, the long-term mean in productivity is not affected by vole population variability. If there is any biological trait contributing to the linearity of the response without any upper bound, then it is the ability of barn owls to breed more than once as compared to Montagu’s harriers, storks or skuas whose reproductive capacity is constrained to one nesting event per year.

Like Hušek et al. [5], we observed a positive relationship between the degree of synchrony (correlation) and population variability of voles. However, because they interpreted the correlation as the strength of the breeding response, this observation is in conflict with Jensen’s inequality theory for concave curves, which predicts the opposite [4]. This discrepancy was then explained by unusually high Taylor’s exponent *b* of about 4 to 6 inducing a strong positive relationship between voles’ temporal variability and population mean. Our results suggest that the high Taylor’s exponent *b* is not a specific feature of the common vole population variability. Instead, it may be the result of data handling procedures which reduced the original amount of variation. In our data, it was due to detrendization. In vole data used in Hušek et al. [5], it was the reconstruction procedure. Their continuous data came from Tkadlec et al. [44] and were derived from annual pest survey maps showing four abundance categories for the common vole. Through this process, a large portion of temporal variation in vole numbers was lost, particularly in districts with low vole’s population densities. These districts are characterized by high proportion of forested areas which are suboptimum habitats for the common vole. Incidentally, it is just these low-density districts that were analysed in the white stork study. Likewise, the elimination of the trend in our study reduced the variance in log population means. Because *b* from Taylor’s power law is a standard regression coefficient computed as Cov (log (Var), log (Means))/Var (log (Mean)), any reduction in Var (log (Mean)) leads inevitably to an increased *b*. Hence, our study adds to a long-term search for situations in which Taylor’s exponent reaches values of *b* > 2 [45].

Surprisingly enough, the shift in *b* due to detrending does not seem to be the cause of increased synchrony of stork productivity with voles’ population variability. As demonstrated by our results, we identified high synchrony in both approaches, suggesting that it is not related to the detrending procedure. As an alternative explanation, we propose that owls in high vole variability areas are more specialized on the common vole which in turn reduces their diet breadth, especially in high-density years. As a result, a stronger ecological signal conveyed by more variable vole dynamics translates into consumer’s productivity dynamics more precisely, thereby resulting in higher correlations between both dynamics. Another feature contributing to the stronger correlations in more variable districts is that higher vole population densities are measured more precisely. For population densities above 1000 burrow entrances per hectare the relative sampling error falls below 10% [46] which, in general, is a desired level of precision for most population measurements [47].

We found insufficient evidence for an increased strength of the productivity responses to more variable vole populations. In fact, our evidence based on AICc did not allow us to discriminate between the models with and without effect. In particular, the approach with detrended data was a better fit than that without it. Surely, detrending non-stationary data does have the potential to shape the outcomes of time series analyses leading us to draw different ecological inferences. Even if real, the effect of variability seems to be quite small and thus may perhaps require much larger sample sizes to actually prove it. However, even if so, it would be very difficult to interpret the effects of the common vole population variability in biological terms. In areas with a broader range of alternative prey and less dependence on voles, barn owls may respond not only less precisely to changes in vole numbers but also less strongly, thus eliciting no adaptive explanation.

By focussing on the barn owl–common vole system, we showed how the pulsed resource can influence the dynamics in the consumers’ reproductive output by bringing them into a close synchrony with the prey. From the life history perspective, it might be more insightful to examine the consumer responses in systems with a curvilinear relationship between productivity and pulsed resource variability which can facilitate testing more specific predictions (e.g., [5]). Furthermore, comparisons of responses in vole-eating consumers with different levels of vole specialization may also help us to better understand not only consumers’ life histories but also the dynamics of such interactive systems.

## Supporting Information

S1 Fig Linear trends (dashed line) in log-transformed autumnal vole numbers (solid line) for ten districts of the Czech Republic. With the exception of one district (ZN), the intercept-only models performed better than the models with time (the difference in AICc > 2).
(EPS)

S2 Fig The relationship between barn owl productivity responses and vole population means in ten districts of the Czech Republic. The upper panels show the degree of synchrony between the barn owl productivity and vole population means using the non-detrending (a) and detrending approach (b). The lower panels show the strength of the productivity response to vole population means without detrending (c) and with detrending (d). The dashed lines indicate 95% confidence intervals for the regression line.
(EPS)

S3 Fig The effects of direct density dependence on synchrony (a, b) and strength of productivity response of barn owls (c, d) using non-detrended (a, c) and detrended data (b, d). The intercept-only models performed better or equally well as the models containing direct density dependence.
(EPS)

S4 Fig The effects of delayed density dependence on synchrony (a, b) and strength of productivity response of barn owls (c, d) using non-detrended (a, c) and detrended data (b, d). The intercept-only models performed better or equally well as the models containing delayed density dependence.
(EPS)

S5 Fig Linear trends (dashed line) in logtransformed autumnal vole numbers (solid line) for ten districts of the Czech Republic. Except the district ZN, the intercept-only models performed better the model with time (the difference in AICc > 0).
(EPS)

S6 Fig The relationship between barn owl productivity responses and vole population means in ten districts of the Czech Republic. The upper panels show the degree of synchrony between the barn owl productivity and vole population means using the non-detrending (a) and detrending approach (b). The lower panels show the strength of the productivity response to vole population means without detrending (c) and with detrending (d). The dashed lines indicate 95% confidence intervals for the regression line.
(EPS)
